# The linguistics of schizophrenia: thought disturbance as language pathology across positive symptoms

**DOI:** 10.3389/fpsyg.2015.00971

**Published:** 2015-07-16

**Authors:** Wolfram Hinzen, Joana Rosselló

**Affiliations:** ^1^Institució Catalana de Recerca i Estudis AvançatsBarcelona, Spain; ^2^Department of Philosophy, University of DurhamDurham, UK; ^3^Department of Linguistics, Grammar & Cognition Lab, Universitat de BarcelonaBarcelona, Spain

**Keywords:** schizophrenia, language, thought, formal thought disorder, hallucinations, delusions, self-disturbance

## Abstract

We hypothesize that linguistic (dis-)organization in the schizophrenic brain plays a more central role in the pathogenesis of this disease than commonly supposed. Against the standard view, that schizophrenia is a disturbance of thought or selfhood, we argue that the origins of the relevant forms of thought and selfhood at least partially depend on language. The view that they do not is premised by a theoretical conception of language that we here identify as ‘Cartesian’ and contrast with a recent ‘un-Cartesian’ model. This linguistic model empirically argues for both (i) a one-to-one correlation between human-specific thought or meaning and forms of grammatical organization, and (ii) an integrative and co-dependent view of linguistic cognition and its sensory-motor dimensions. Core dimensions of meaning mediated by grammar on this model specifically concern forms of referential and propositional meaning. A breakdown of these is virtually definitional of core symptoms. Within this model the three main positive symptoms of schizophrenia fall into place as failures in language-mediated forms of meaning, manifest either as a disorder of speech perception (Auditory Verbal Hallucinations), abnormal speech production running without feedback control (Formal Thought Disorder), or production of abnormal linguistic content (Delusions). Our hypothesis makes testable predictions for the language profile of schizophrenia across symptoms; it simplifies the cognitive neuropsychology of schizophrenia while not being inconsistent with a pattern of neurocognitive deficits and their correlations with symptoms; and it predicts persistent findings on disturbances of language-related circuitry in the schizophrenic brain.

## A Novel Perspective on Language and Thought in Schizophrenia

On a common view, language is a vehicle for communication, grounded in pre-existing thought, which provides its content. On this view, delusional symptoms like a person’s belief that he is Jesus Christ are typically classed as disturbances in ‘thought’ rather than language: language merely ‘reflects’ an underlying disturbance in the thought process, without being an aspect of its etiology^1^. If language is merely a tool, and this tool is detached or broken, we would then expect that thought could stay the same, and *vice versa*. Global aphasia, where core cognition can seem surprisingly normal may illustrate precisely such a predicament (Varley, 2014; but see Baldo et al., 2010). If aphasia is a language disturbance, not a fundamental cognitive one, schizophrenia could then be regarded as the reverse case, in line with the clinical impression that aphasia-like language disturbances are not characteristic of either schizophrenic speech or comprehension, and the empirical finding that bad performance on aphasia-type test batteries is a function of the general intellectual impairment seen in patients with schizophrenia rather than a specific neuropsychological deficit (Oh et al., 2002).

Bleuler (1911, p. 121) classically confirms this perspective when he notes that the primary symptoms of schizophrenia ‘find their expression in language,’ but ‘here the abnormality lies not in language itself, but in what it has to say.’ Unlike in aphasia, that is, where patients struggle to communicate normal thoughts that others would articulate in normal sentences, in schizophrenia the meaning itself is distorted. Yet it is clear that we cannot separate ‘what language has to say’ (meaning) from ‘language.’ More specifically, it is an inherent aspect of language that it conveys content of a particular *kind*, which carries information about the world and from which we can *learn*. If language stops conveying such content, as in delusional statements that cannot be true (‘I came to earth in a cosmic bubble’), or disordered speech (‘A conclusion is my French professor’), language *loses* its function of carrying and conveying such content. If having and conveying such content is an inherent aspect of language, such loss therefore *is* a disorder of language, though crucially not an aphasic one. Bleuler (1911) himself didn’t fail to document a fundamentally altered relationship that some of his patients had with language, to the point of them developing artificial languages (*Kunstsprachen*) used as if they were normal ones. Moreover, his essential experimental methods were word association experiments carried out with his assistant C. G. Jung, who theorized that ‘words are really something like condensed actions, situations, and things. [They are] linguistic substitutes for reality’ (Jung, 1910, p. 223) – in short, the specific currency of human thought.

Comparative studies of cognition and communication across species confirm an explanatory gap between linguistic and non-linguistic cognitive and communicative contents (Hauser, 1996; Penn et al., 2008; Tomasello, 2008). Some philosophers (e.g., Davidson, 1982, 2004) deny the very applicability of the term ‘thought’ to non-verbal species. Even if we don’t follow them, language correlates with forms of thought, history, and culture that are highly distinctive. It plays a crucial role in cognitive development (Vouloumanos and Waxman, 2014). Where language does not develop normally, thought is altered as well, as in children on the autism spectrum (Eigsti et al., 2011), or it radically impoverishes as in language-less adults (Schaller, 1991) or deaf children deprived of a sign language (Humphries et al., 2014). In the context of hominin evolution, a radically different mind-set separates our own version of *Homo* from all other species in this genus, in which language is absent or uncertain (even if speech was present). According to Tattersall (2008, 2014), language is the most likely cognitive principle that transformed pre-sapiens cognitive phenotypes into their modern human variety, *re-configuring* the hominin mind rather than merely *expressing* one that pre-existed the arrival of linguistic communication. Supporting this perspective, Hinzen and Sheehan’s (2013) recent ‘Un-Cartesian’ linguistic framework (Sheehan and Hinzen, 2011; Arsenijevic and Hinzen, 2012; Hinzen, 2014, 2015; Martin and Hinzen, 2014) provides a language-based account of human-specific forms of thought, reference, and selfhood centered on grammar. In the wake of language, new diseases affecting this new mind could then have arisen as well, with schizophrenia as a potential example: the ‘price we paid for language’ (Crow, 1997, 2007; see also Kleist, 1914).

The un-Cartesian linguistic framework goes further than recent foundational views that grant language a ‘supra-communicative’ function, over and above its function as a tool of interpersonal communication (Carruthers, 1996; Clark, 1998). On Clark’s (1998) view, language functions as a technology to ‘enhance’ cognition and facilitate thinking through verbal assistance, aiding mental computation much like other external artifacts such as sextants, compasses or maps do. Inventing a compass or a map, however, is something that a creature does that already has a modern human mindset. What is to be explained is a difference in the fundamental *cognitive type* that characterizes modern humans, putting a form of rational thought in place that we do not see in non-human species, including extinct human ones. The question that un-Cartesian linguistics addresses is how we obtain this cognitive type, which uniquely invented language and started communicating linguistically^2^. Its central claim, that the human-specific form of rational thought and language arose together, makes the prediction that they should also fall together. It follows that schizophrenia could be re-conceptualized as manifesting a breakdown of the linguistic frame of thought and hence that it can be illuminated in linguistic terms.

### The Hypothesis

The hypothesis of this article is that schizophrenia is a breakdown of how language configures thought in the normal brain, viewed against an un-Cartesian background theory of what language is. Language circuitry in the brain is disturbed, resulting in forms of thought that cannot be shared anymore and lose objectivity, including thoughts about other minds, leading to a breakdown of normal social cognition and communication that depend on the linguistic frame of thought being intact.

### Four Predictions

(1) Most fundamentally, language should illuminate cognitive change seen in symptoms, which should not only have linguistic interpretations, as already argued by Crow (1997), but involve a malfunctioning in core linguistic variables that are key to what ideas we can communicate in language. This malfunctioning should not be seen in superficially similar conditions such as ‘fluent’ aphasia, when and as long as cognition in these conditions is normal or changed in different ways than it is in schizophrenia. In short, across symptoms and conditions, linguistic and cognitive profiles should *match*.
(2) Studies of the language of schizophrenia after Chaika (1974), (Rochester and Martin, 1979; Morice and Ingram, 1982; Wykes and Leff, 1982; Oh et al., 2002; Covington et al., 2005) have tended to *use* linguistic theory as a way of formalizing and identifying anomalies in schizophrenia speech rather than as an explanatory framework. By contrast, the un-Cartesian linguistic framework generates the prediction that the linguistic anomalies we will find in such speech specifically concern the ways in which, according to this framework, grammar mediates referential, and propositional forms of meaning. A very specific prediction is that *the more forms of reference are mediated grammatically, the more they should manifest anomalies/impairment* (e.g., the use of deictic and definite noun phrases should be more impaired/anomalous than the use of non-definites).
(3) The more language-dependent a neurocognitive variable is, the more it should be affected (see Section “Language and the Cognitive Neuropsychology of Schizophrenia”).
(4) The neural correlates of core symptoms – and even of key cognitive variables including ‘theory of mind’ (ToM) – should be part of the language network.

### The Theory

**FIGURE 1:**
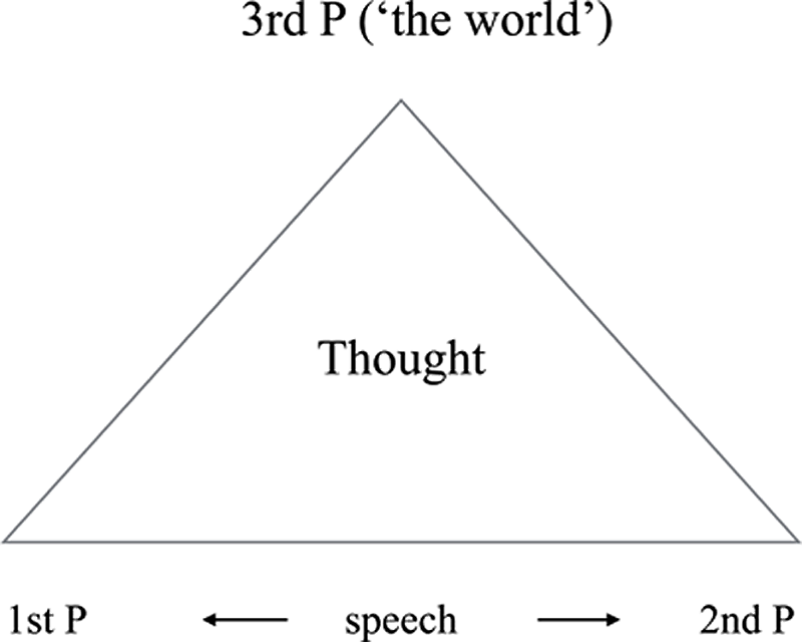
**The deictic frame of speech and thought.** A grammatical 1st Person, addressing a 2nd, talks about an object or event in the world (the ‘it’ or 3rd Person) expressing a thought.

Language is an *integrative system*: therein lies its special status among all other neurocognitive variables. In any utterance we ever make, all of the cognitive functions come together in a coherent way: speech, thought, affect, perception, memory, attention, planning, etc. If language falls apart, ‘loosening of associations’ in Bleuler’s (1911) sense – i.e., the disintegration of the ‘psychic functions’ – should therefore result. More specifically, we can depict language as a triangle having three essential corners: speech production, speech perception, and content (we cannot but talk about the world). None of the corners are independent of any of the others [hearing and speaking go together as capacities (Menenti et al., 2011), and there is meaning in both, inherently]. Moreover, in speech, the speech agent identifies himself as the subject of the speech act in the grammatical 1st person, and as talking to a speech-perceiving agent identified in the grammatical 2nd person. In this sense, all of our utterances are embedded under a silent ‘*I* think/say to *you* that.’ Moreover, all such talk is about the world, the grammatical 3rd (or non-) Person (see **Figure 1**).

**FIGURE 2:**
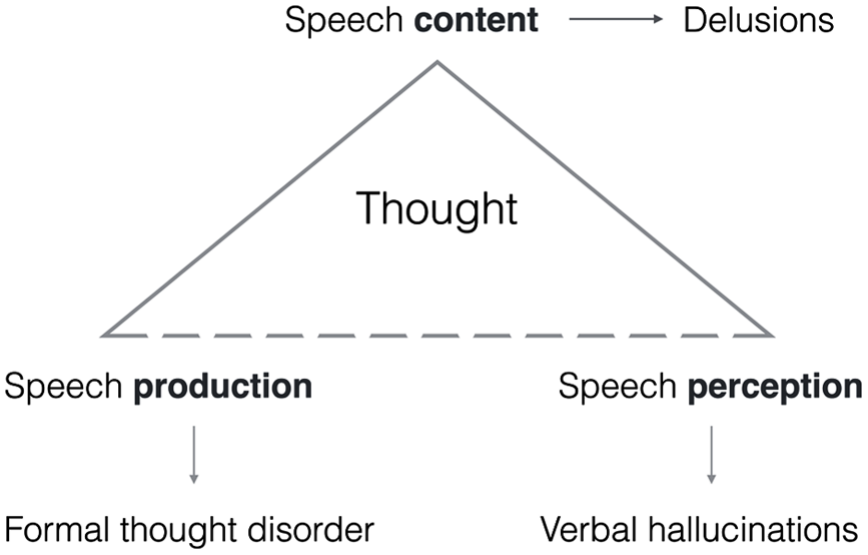
**The three positive symptoms derived as a breakdown of the linguistic frame of thought.** Human language organizes speech perception, production, and content as the co-dependent angles of a triangle. Depending on which corner is affected most, symptoms result.

Depending, then, on which corner is disproportionally affected by the disease process (perception, production, or content), creating an imbalance in the normal language function, we can give a linguistic characterization of the three core positive symptoms according to current DSM-5 criteria in the following way (**Figure 2**):

• A disorder of speech perception leading to a conflation of thought and speech, as in *Auditory Vocal Hallucinations (AVHs)*.
• A disorder of speech production leading to disordered speech without feedback control, as in varieties of *Formal Thought Disorder (FTD)*.
• A disorder of content formation leading to distorted meanings that cannot be true as in *Delusions*.

### The Evidence

We currently test predictions (1)–(2) above^3^. Current support for the hypothesis and theory firstly comes from the independent un-Cartesian linguistic theory (cf. Section “How can Language Illuminate Psychosis?”), which argues empirically that a propositional and referential capacity in humans is based on language, depending on grammar (which crucially includes a threefold Person distinction) as its essential principle of organization. A breakdown of language thus viewed predicts the loss of a propositional and referential capacity. This loss is virtually *descriptively equivalent* to what we see in positive symptoms as described in Section “Linguistic Dimensions across Positive Symptoms.” These can thus not only be given linguistic characterizations, in which core linguistic variables identified in the linguistic theory figure, but these descriptions provide a *mechanism* for symptom formation. The connection between the neurocognition of schizophrenia (i.e., language) and symptoms now becomes immediate. In contrast, relating general or specific neurocognitive *deficits* (say in memory or executive function) to the ‘excesses’ of functions that the positive symptoms represent (especially hallucinations and delusions) has been a persistent problem (McKenna, 2007, Chap. 8–9). The inherent involvement of language in human intelligence makes our proposal nonetheless consistent with a pattern of general intellectual impairment found in patients with schizophrenia, which is less clear in Bipolar Disorder (Mann-Wrobel et al., 2011; Bourne et al., 2013; Crow et al., 2013; Bora, 2015; but see Kuswanto et al., 2013), where language, though not perhaps in all subgroups, may be affected less and differently (Morice and Ingram, 1982; Andreasen and Grove, 1986). It is also consistent with a pattern of specific impairments found in neurocognitive variables such as declarative memory or ToM, neither of which is arguably language-independent (see further Section Language and the Cognitive Neuropsychology of Schizophrenia).

We review four decades of findings on the language of schizophrenia in Section “The Language Profile of Schizophrenia against our Predictions.” The pattern of these findings, we suggest, is strongly consistent with our predictions. In Section “Language in the Schizophrenic Brain,” we briefly review the neural correlates of schizophrenia in the light of our hypothesis, and again suggest that findings on disorders in speech perception and comprehension areas as well as ToM areas are consistent with our account. Due to space limitations, we do not discuss negative symptoms in this paper.

## Linguistic Dimensions across Positive Symptoms

According to current DSM-5 criteria, a diagnosis of ‘schizophrenia’ requires the presence of at least one of the following three ‘positive’ symptoms (DSM-5 295.90, p. 99):

(1) Hallucinations
(2) Delusions
(3) Disorganized speech

A long-standing orthodoxy suggests that *none* of these syndromes inherently relate to language^4^.

### The Linguistic Profile of FTD

In FTD, a connection with language is obvious: DSM-5 refers to it as ‘disorganized thinking,’ which is ‘typically inferred from the individual’s speech’ (p. 88). Such speech can be productive (no ‘poverty of speech’), and patients can be fully cooperative communicators. Yet however much speech is produced, the listener has difficulties identifying what is actually being ‘said’ (‘poverty of content’). In another classical profile, patients are clinically said to exhibit ‘loss of goal,’ ‘derailment,’ and ‘tangentiality’. One example of each, from McKenna and Oh, 2005, p. 34, are given in **Box 1**):

**Two examples of Formal Thought Disorder (from McKenna and Oh, 2005, p. 10 and 43).**

Q. How do you like it in hospital?
A. Well, er not quite the same as, er don’t know quite how to say it. It isn’t the same, being in hospital as, er working. Er the job isn’t quite the same, er very much the same but, of course, it isn’t exactly the same.
Q. How are you?
A. ’To relate to people about new-found talk about statistical ideology. Er, I find that it’s like starting in respect of ideology, ideals change, and ideals present ideology and new entertainments new, new attainments. And the more one talks about like, ideal totalitananism, or hotelatarianism, it’s like you want new ideas to be formulated, so that everyone can benefit in mankind, so we can all live in our ideal heaven. Presumably that’s what we still want, and with these ideas it can be brought about. I find the it’s like a rose garden.’

In poverty of content, phrasal and even sentential structure can seem normal, but the thought carried by language is hard to pin down. Thus in the second example, an initial topic is not addressed, a new topic is vigorously set, but the speaker derails, and the discourse is carried forth more on the basis of lexical associations than propositional meaning. Overall, no point or message emerges.

Chaika (1974) provided the first sustained linguistic analysis of such speech after the onset of modern linguistics in the 1950s. According to her, the anomalies concern how words are combined into meaningful sentences and discourses according to linguistic rules, in particular the ability to organize discourse according to sentence topics, hence grammatically, insofar as the notion of topic correlates with that of the grammatical subject. By consequence, words start appearing in strange grammatical contexts with no stable sound-meaning associations, (e.g., the clanging association in ‘I had a little goldfish too, like a clown. …Happy Halloween down’), which also leads to topic losses and ‘derailment,’ and unresponsiveness to questions (tangentiality). Chaika (1974) rejected the restriction of the single sentence as a unit of linguistic analysis, taking the discourse to be the relevant unit. Nonetheless, individual sentences themselves can also become fragmented and ungrammatical (e.g., ‘I’m be puped tall letter I’m write to you,’ from Chaika, 1982). Moreover, sound combinations can be produced that cannot be identified as words (e.g., ‘He still had fooch with teykrimez,’ from Chaika, 1974), have invented derivations (e.g., ‘plausity,’ ‘puterience,’ ‘amorition,’ from Chaika, 1982), appear in unusual compounds (e.g., ‘night-illuminating object’ for ‘lamp’), or no static meaning (McKenna and Oh, 2005, p. 82). Typically, speakers of such speech are not aware of such abnormal production, suggesting impairment in meta-reflexive or self-monitoring capacities: a feedback loop from production to thought is missing.

The intrusions of semantic (Pomarol-Clotet et al., 2008) or phonological word-level associations inappropriate to the context in which the words appear grammatically are nicely demonstrated in the following example adapted from Chaika (1974), where use of the sound /bill/ as appearing in its first grammatical context requires processing it as a person-denoting proper name, after which the same sound is re-grammaticalized as a verb denoting an action (as in ‘bill and coo,’ i.e., making love), and further associated with a common noun denoting a bird’s body part:

‘My mother’s name was Bill. [pause] [low pitch, as in an aside, but with marked rising question intonation]… and coo? St. Valentine’s Day is the official startin’ of the breedin’ season of the birds. All buzzards can coo. I like to see it pronounced buzzards rightly. They work hard.’

In a production like ‘A conclusion was a French professor,’ uttered by one of the FTD patients in our study, the system of grammatically categorizing ideas seems to have broken down (a conclusion needs to be grammatically a sentence, not an indefinite Noun Phrase like ‘a French professor’).

Over time, Chaika’s (1974) essential claim that there is a ‘linguistics of schizophrenia’ distinct from both aphasia and slips of the tongue in normal speech, has held up (Oh et al., 2002; Covington et al., 2005; McKenna and Oh, 2005), and all of the essential characteristics of such speech that Chaika (1974) listed have by now become reliably identifiable as part of standard rating scales such as Chen et al.’s (1996) Clinical Language Disorder Rating Scale (CLANG). Overall, there seems to be no way to identify and characterize this syndrome *independently* of the abnormal linguistic patterns in which it consists and as which it is diagnosed. Specific linguistic processes and principles of organization – e.g., derivational morphology, phrases vs. sentences, referential phrases, proper names vs. common nouns, etc. – are involved in it, which have to come together in the right way to generate the clinical impressions of ‘emptiness’ and ‘disorganization’ that we see.

### The Linguistic Profile of Hallucinations

Hallucinations are defined in DSM-5 as ‘perception-like experiences that occur without an external stimulus’ (DSM-5, p. 87). Auditory ones are identified as the most common. These in turn prototypically take the form of voices, whether familiar or unfamiliar. These do not prototypically grunt, bark or whistle, but *talk*, using words and (usually short) sentences, and hallucinations in this sense take an auditory *verbal* form (A*V*Hs). AVHs therefore are inherently a *linguistic experience* and hence involve an *anomaly of speech perception* by definition. This speech further has contents that ‘are perceived as distinct from the individual’s own thoughts’ (ibid.), which is to say that they are perceived as the thoughts of what is, from the viewpoint of the voice hearer, a grammatical second (‘you’) or third (‘he’/’she’) person, while the voice hearer in turn is a second or third person from the viewpoint of the voices. This entails a loss of first-person ownership of the patient’s self-generated thoughts, which we earlier identified as the implicit subordination of every thought or utterances under the phrase ‘I think that.’ To illustrate, suppose an example of the thought in question is (1):

(1) I am weak.

Then there is a transition from this thought, to the thought that would in mental health be expressed by another person’s utterance of the form in (2), which is now what the voice is heard to say (‘*He* thinks/says that’):

(2) He is weak.

We will call this a *deictic shift*, specifically from the 1st to the 3rd (or else to the 2nd Person). While the words ‘I’ in (1) and the word ‘he’ in (2) refer to the exact same person, i.e., the voice hearer, the thought as expressed in the two utterances has crucially become a different one and the identification ‘he = I’ is not made by the voice hearer, even though he is perceiving what are effectively his own thoughts. In delusions the identification *is* made: ‘he’ becomes ‘I,’ leading, e.g., from ‘He is Jesus’ to ‘*I* am Jesus’. This seems to indicate a broader pattern of the loosening of deictic anchoring to which we return.

Again these initial observations suggest that not only is language a defining feature of *AVHs*, but its linguistic profile involves specific grammatical distinctions and pronominal patterns, without which the phenomenon would not be what it is. In line with this, language plays a crucial role in Bleuler’s (1911, pp. 79–84) extensive discussion of hallucinations. He notes that elementary auditory hallucinations (e.g., hearing shooting or the wind blowing) were relatively rare, and if they occur they are normally interpreted as involving *reference* to the patient (e.g., the shooting occurs to rescue him), hence they really represent what are now termed referential delusions. Crucially, reference is nothing we can *hear*. As we argue in Section “How can Language Illuminate Psychosis?,” it is a *linguistic* category inherently. The most common auditory hallucinations, Bleuler (1911) further notes, are those of language: ‘music is rarely heard’^5^. For verbal hallucinations, moreover, *audition* is not actually essential, as is now widely documented (McCarthy-Jones et al., 2013): many so-called ‘voice hearers’ ‘cannot tell whether they hear the voices or whether they only have to think them; they are “lively thoughts,” which are nonetheless still called voices by the patients themselves; then again they are “loud thoughts,” “toneless voices” (…)’ (Bleuler, 1911, p. 90). What precisely cuts across these phenomenological differences is that *language* is perceived (whether or not sound is), in the sense that the thoughts in question have a linguistic *articulation*, come with a *content* that is given by this articulation, and that such thoughts/voices often appear as acts of linguistic *communication* to the recipient (even though the communicative situation is unusual).

These acts are not dismissed by patients as curious auditory accidents, but, e.g., as *meaningful commands* (‘Go into the water’), which are often *followed* by them, turning into the thought ‘I go into the water,’ just as for a person who gives instructions to herself using self-talk (‘Climb this mountain!,’ ‘Give her a kiss now,’ etc.). In this regard the voice hearer is confused as to whether the thought ‘he says I should do X’ really is the thought ‘I should do X.’ There is a Person confusion in addition to a shift, which in and of itself would not entail the phenomenology of AVHs. Perceiving speech of other people normally simply means that we recognize them as individuals like ourselves, who have their own bodies, 1st-personal narrative histories and beliefs about the world, and whose commands for actions are just that: things that someone else wants us to do. But voices do characteristically not develop a personal narrative or talk much about themselves in the 1st Person, nor about the world, using the 2nd or 3rd Persons near exclusively (‘you’ or ‘she/he’/‘this loser,’ etc.). In commands like ‘wash your hair’ endlessly repeated, heard speech content cannot be rationalized any more, as it normally can be, and voices cease to be rational speech agents. Nor is such speech appraised as normal speech would be. We may on occasion misattribute speech, but we would not hear the fridge or the bread talking to us. Nor would we wonder why we can hear our relatives talking to us, while we have to travel to them in order for them to do so (Bleuler, 1911, p. 94). Many patients, Bleuler (1911) notes, do not look for explanations for why the voices are there at all.

Lack of rationality transpires at the content level itself, where such speech can appear like a tragic parody of normal language. One voice commands that the patient goes into the water; at the same time she mocks and chides him for doing so (Bleuler, 1911, p. 80). Molly Watson’s voice ‘Take it easy, but remember, it won’t be easy. Nothing will be easy.’ ‘We learn something and we learn nothing’ (Watson, 2015, pp. 6–7). Some voices comment passively and irrelevantly on what patients currently do. Some patients perceive something, and the voice says aloud what it is (‘a wedding’), reflecting an involuntary intrusion of speech into the normal speechless perceptual process. Others say out loud what the patient just thought, or repeat what he says the moment he stops. According to Bleuler (1911), only rarely do voices say who they are, and the patients are typically not interested in this, or know it already; sometimes the patient’s confusion shows in many voices talking at the same time, so that no thread can be followed. Threats and abuses abound, as do commands, which correspond grammatically to reduced clause types whose purpose is not to share information about the world, make assertions, and express thoughts, and develop narrative, but give directives for action. Asking questions (interrogatives), too, is not a voice strength.

There is, then, a speech profile to voices^6^. A distortion of normal propositional language and thought characterizes *both* FTD and AVHs, though in different ways, with a disorder in the interface between thought and speech as the distinguishing feature of AVHs. AVHs not merely represent a (i) disturbance in speech perception, (ii) deictic shifting and Person confusion, (iii) content distortion, and (iv) grammatical narrowing, but also (v) the erasure of a normal boundary between thought and speech, providing another linguistic index for a disintegration of the self, for whose integrity it is essential that one’s 1st Personal thoughts are one’s own and private to oneself. It is a defining phenomenological feature of normal 1st Person thoughts that they are silent: we can voice them, if only by moving our lips, but if we don’t, we don’t hear them. If we utter them and they become overt, they become also different, being necessarily addressed to a grammatical 2nd Person (not ‘I think…’ but ‘I say to you…’). In AVHs, thoughts become perceived as speech when they should be silent ^7^.

### Delusions

Finally, delusions are defined in DSM-5 as beliefs rather than perceptions, which is what hallucinations are. Qua perceptions, the latter are not under voluntary control, while a belief, being rational, *is* typically taken to be, like an assertion: we can *decide* what we say or believe. The beliefs in question are further described as ‘fixed,’ and as held with incontrovertible certainty. These beliefs must be linguistically articulated internally, on the assumption, to which we return, that a belief like ‘I came to earth in a cosmic bubble’ cannot be formed without linguistic articulation. This already entails an anomaly in language, since delusions are beliefs that come with a quasi-perceptual force, which propositions expressed as sentences in language characteristically not only *lack*, but which truth does not *require* (things can be true even if this truth is not evident or forceful).

In line with the anomaly of their incontrovertible certainty, delusions are not contentful but contingently false, like a person’s claim that coffee grows on trees. Instead, they are propositions that are often bizarre and judged by others as being *impossibly true* (‘Doctor, I wear my father’s hair’; ‘I am Jesus Christ,’ ‘The Catholic Church is trying to kill me’). These, moreover, are often generated without much consideration or reasoning – they can simply ‘pop up’ and patients can typically not justify what they believe. Apart from such delusions, there are *referential* delusions such as the belief that gestures, utterances, and other environmental cues refer to the patient. Also included among delusions in DSM-5 are delusions expressing a loss of control over one’s mind or body, e.g., thought withdrawal (one’s thoughts have been removed by an outside force) and insertion (alien thoughts are being put into one’s mind), i.e., Schneider’s (1957) first rank symptoms.

We take the prevailing view to be that none of these delusions are in some essential sense language-related. Yet as noted above, it is part of the essence of language that it carries a content of a particular kind: content, in particular, that can be true even if false, and can, unlike a perception, be negated and reasoned with. Language is not a system to which such content accrues somehow accidentally: content is not something that it ‘also has,’ and could also lack, while staying what it is. In virtue of having a certain grammatical structure – with a predicate attributed to a subject – a sentence *ipso facto* has such content. The most immediate thing to say, therefore, is that in the expression of a delusion such as ‘I am Jesus,’ a predicate (i.e., being Jesus) and a subject (‘I’) are combined in a way that a monstrous propositional content arises. At the word level, ‘Jesus’ is presumably used correctly, but like in FTD, the mistake lies at the *grammatical* level, i.e., how words are related grammatically as subjects and predicates. In particular, while the speaker still uses the proper name ‘Jesus’ correctly to refer to *Jesus* (not Napoleon), he is not using ‘I’ properly to locate himself in deictic space, as a bearer of the property of being Jesus. Where ‘He is Jesus’ would be fine, a shift to the 1st Person has taken place, and ‘I am Jesus’ results. Similarly, where ‘The Mafia is trying to kill *someone*’ is surely true, and ‘the Mafia’ is used correctly, a shift to the first Person takes place here too, resulting in a falsehood ‘The Mafia is trying to kill *me.*’

We do not think we learn anything about the world from such delusional statements, which do not trigger any criminal or religious investigation. Unlike all normal assertions, we take them to inform us about the (pathological) thought of the speaker *only*. The structure of meaning – the triangulation – has thus fundamentally shifted^8^. A dysfunction of the propositional function of language therefore is not restricted to FTD and AVHs but concerns delusions as well, with one difference: in severe thought disorder, the referential function of language is impaired at all levels; in delusions, the propositional capacity is sufficiently intact to nail down at least one referent – the Mafia, or Jesus – but it then derails at the grammatical level, where no propositional contents relating to the world are obtained. Bleuler’s (1911) primary symptom called ‘ambivalence,’ too (patient utters ‘I am a human being like you’ and ‘I am not a human being like you,’ now falls into place as an instance of the same problem with the propositional function of language: in delusions, propositionality is resolved in favor of perception, and a sense of truth is lost; in ambivalence, the question which proposition is true cannot be resolved.

Against this account, Frith (1992, p. 95) adopting the standard view that delusions have nothing to do with language, asserts that delusions are a ‘private’ matter that, *unlike* FTD, cannot be studied objectively and ‘directly.’ But the disorder that delusions represent is exactly as overt and objective as that of FTD: delusions are linguistically articulated thoughts, even if not pronounced, and pronounced they typically are. They are not known (and could not be known) to be held in some ‘private’ mental space, while never appearing in overt language. The essential difference between FTD and delusions is that in the latter case, contents can still be identified but are judged to not possibly be true, while in the former no contents may even be discernible. Either way, the problems concern meaning generated at a grammatical level.

Frith’s (1992) statement thus implies a Cartesian thought-language distinction, where the normally inherent integration of the two functions has become a contingent one. We could insist for metaphysical reasons on a Cartesian distinction between ‘thought’ viewed as something non-linguistic, and ‘language’ as merely an expressive tool. Yet in general, the exact identity of a thought depends on the constituents and relations among the parts that it contains, which we *see* in the sentences expressing them. We would therefore have to stipulate the existence of exact non-linguistic ‘mental’ equivalents of these constituents and relations at the level of ‘non-linguistic thought’. But this extra step seems to reap no explanatory benefits and the same problem now arises: why are the mental equivalents of ‘I’ and ‘Jesus’ combined in such ‘non-linguistic thought’ as subject and predicate in a fashion that a proposition arises that cannot be true, yet is taken as true with quasi-perceptual force?

As in AVHs or FTD, the linguistic elements involved in delusional statements are again specific. Thus ‘Napoleon is Hitler’ is not a likely delusional statement that a clinician would expect to find; nor are ‘The Mafia is trying to kill Obama,’ ‘German towns are beautiful,’ ‘I will be Jesus,’ or ‘I think I am Jesus.’ These are *grammatically* of the wrong types^9^. Instead, typical examples of delusions involve the grammatical 1st Person combined with a 3rd person predicate, in a non-embedded sentence, often copular, typically in the present tense or with an atemporal meaning. If changing the grammar entails changing the status of a sentence as a delusion, grammar *is* essential to its status of a delusion, and propositional delusions have a linguistic identity not contingently, but necessarily.

In the mouth of a normal speaker, moreover, the statement ‘I am Jesus’ means something different: it could be a joke or metaphor, or it could mean that the speaker is the Jesus figure in a play. The schizophrenia patient says none of these things, but rather *fixes his 1st Person identity via a 3rd Person description*, which is impossible in mental health, where no such description *ever* accounts for our 1st Person identity as *selves*: *any* descriptive properties we have (‘I am X’), we can in principle also lose (‘I found out that I was switched as a newborn and baptized a Y’). For the patient with schizophrenia, not being Jesus would be for him not to exist. When ‘I am Jesus’ expresses a delusion, therefore, the sentence is not used with its normal meaning: language *changes*. Again there is a Person shift involved.

Some of the same linguistic elements show up in the case of referential delusions, where, e.g., utterances or other signs are misinterpreted as being conspirationally directed at the patient, who thereby mistakenly takes himself to be grammatically a referent in the 2nd or 3rd Person, from the point of view of the speaker: acts of reference and communication about the patient are assumed to exist, where there are none. The communication of such content is a linguistic matter, even if non-linguistic elements (say, white T-shirts, which the patients takes to indicate a conspiracy against him) can be used to code for it. As argued in the un-Cartesian framework, reference *is* the prime function of language. Even referential gestures such as declarative pointing are closely correlated with language in human development (Butterworth, 2003; Cartmill et al., 2014). In the case of referential delusions, this referential arrow is fictitiously pointed from a 3rd person to the patient’s 1st.

Delusions of *thought control*, too, involve confusions in the function of the system of grammatical Person. All thoughts generated have to be deictically anchored in one of the three grammatical persons: they are mine, yours, or a third party’s; or else they are viewed simply as facts (nobody’s thoughts, but an aspect of reality). When this species-specific deictic frame is disturbed, we predict the Schneiderian first rank symptoms that DSM-5 subsumes under delusions, in line with Crow (2010): thoughts are generated, but the patient doesn’t know anymore whose thoughts they are, which now float free of their deictic anchors. There is direct connection here with AVHs: in delusions of thought control, it is thoughts that lose their anchoring in deictic space; in AVHs, it is utterances; and as noted the dividing line between them is not sharp, as an acoustic element need not be central.

## How can Language Illuminate Psychosis?

We have tried to lay bare the linguistic dimensions involved in positive symptoms, addressing our first broad prediction and suggesting in each case that these dimensions are inherent. Yet even granting that language matters, *why* should it do? What is it about language that makes it an essential neurocognitive variable involved? Can linguistics illuminate psychopathology *apart* from providing formal tools for analyzing anomalies in language production and perception that we see? We answer that a theory of psychosis qua ‘reality distortion’ requires specification of a principle – some unified system – from which a sense of ‘reality’ in human cognition *derives*. Such a sense – the basis of science and rationality – is in part human-specific. It does not come from perception, nor from logic alone, assuming that the latter already presupposes possession of a notion of truth, which in its human-specific form is arguably language-dependent (Davidson, 1982, 2004; Hinzen and Sheehan, 2013, Chap. 9). Within linguistically articulated thought, moreover, *words* in isolation cannot be true or false; nor can *phrases* be when seen in isolation, such as ‘the car,’ which taken as such is meaningless. Only as part of a full sentence like ‘The car I saw was red,’ uttered in context by an embodied being, it carries meaning involving referential expressions picking out specific objects and events. Language by its nature, moreover, is *shared*, crossing *between* minds and integrating them with a reality independent of thought. Not being an aspect of individual cognition confined to a single person’s brain, language seems uniquely placed to explain how, among speakers sharing a language, a sense of a shared, objective reality can arise.

This also follows if we accept Bleuler’s (1911) conception of schizophrenia as based on the ‘disorder of associations,’ which is characterized as a disintegration of all the ‘psychic functions’ in their normal interconnections: thought, speech, memory, perception, personhood, affect, etc. For it is precisely in every single *utterance* we make, and only in language, that *all* of these cognitive functions come together. Language unlike other neurocognitive variables is an *integrative system* par excellence, and therefore a prime candidate for the disintegration in question (‘splitting,’ *Spaltung*). The unity of the person or self, moreover, which Bleuler (1911) saw affected, is normally rooted in the person’s 1st Person conscious self. ‘1st Person,’ however, is a *grammatical* notion, defined through its contrast with the grammatical 2nd and 3rd Person. Explications of what selfhood or ‘first Person subjectivity’ are, standardly invoke reference to oneself in the grammatical 1st Person, and we know of no account of such self-reference that does not explicitly or implicitly invoke the 1st Person pronoun, a crucial and universal element of linguistic organization. Invocations in phenomenological psychiatry of such terms as ‘Ichstörung’ (German for self-disturbance, Spitzer, 1988; Mishara et al., 2014) or ‘Me-ness’ (Sass and Parnas, 2003; Parnas et al., 2005; Sass, 2014) are cases in point^10^. It is plausible that our intuitive notion of self is *layered* and not all forms of selfhood require language. Yet a full human self, as the subject of creative thought in the 1st Person and with a content of thought that is objective and shareable, depends on language development. It is language that gives us a self-narrative, a past and history, a shared culture, and imaginary worlds to contrast the actual world with. A *purely* phenomenological account of self-disturbance, which were to see human experience as taking place in a completely pre-linguistic experiential space, with language as only a secondary method of ‘translating’ its contents for others, would be naïve^11^.

Consider an example of a ‘self-disturbance’ reported as: ‘I often feel that it is not I who is thinking.’ Could a structurally rich and meticulously articulated thought such as this reflect ‘experiences’ that are as such non-linguistic and prior to language, yet described correctly by this sentence? How could the ‘experiences’ *be* correctly described, if they did *not* exhibit the structural distinctions the sentence encodes? Having the experiences in question takes *thought*, it is not like seeing a flash of red. A person of whom the above sentence is true is richly reflecting on his or her experiences as and when these occur, activating the conceptual network (Binder et al., 2009), and combining its element into meaningful thoughts. The sentence in question could not be true, in particular, without such a person at the moment of such experiences having a *notion* of ‘thought’; or a sense that a thought can be *mine* or *yours*; or that a thought is not a *fact*. A dysfunction in a system whose normal cognitive function it is to enable such thinking predicts that uncertainties like the above will arise^12^.

In the above instance such an uncertainty is articulated correctly and grammatically: external language functions sufficiently so as to *capture* a prior self-disturbance, which the patient asserts and hence does take 1st Person ownership for. But we argue that the language system does not do so at the moment when the experience occurs: here the deictic distinctions slip. In FTD, language disintegrates further so as to lose the power even of *post hoc* description; in delusions, the referential function of language, which connects concepts to objects in the world, is partially kept (the Mafia exists), yet the objects identified lack the properties ascribed to them (the Mafia does not try to kill me). These various distortions reveal health risks that uniquely arise for a mind endowed with a language capacity. Thought carried out in language is not stimulus-driven (Chomsky, 1959), being based rather on internally stored concepts that can be activated at will, as opposed to percepts, which depend on the presence of a stimulus for their activation. Once creativity is there, the risk is that it over-produces, creating delusions or confabulations, which erase a boundary between personal and non-personal truth that language normally maintains. The normal case, where personal thoughts freely created out of concepts can be shared in language and reflect an objective world, is thus a fragile equilibrium. We see it shaken where reference only partially functions, while predication is lost, or propositional content becomes inaccessible to a mind altogether.

Understanding this fragility requires invoking the one system we know where such contents exist. Concepts are most directly associated with *words*: for every concept there is a word (though maybe not in all languages), and with every word goes a concept. Yet lexical concepts alone – HOUSE, MAN, GO, MOUSE, DOWN – give us no content (or thought) in the above sense. A principle of combination is needed. But there is no known principle of combination of a kind that can productively generate a potential infinity of thought with the relevant kind of contents, than grammar. Grammar *is* the system that makes words become nouns and verbs, gives them grammatical roles, turns them into subjects and referential expressions, or else predicates exhibiting generality. From the combination of subjects and predicates in this sense, propositions arise, which are what can be true or false. The function of grammar is thus not to add to our stock of concepts but to make them referential, moving from MAN to ‘the man,’ where the former cannot refer to an individual man but the latter can. Referentiality comes in a number of specific forms, thus we can refer *generically* or *indefinitely* to the world (‘men are deceivers,’ ‘a man entered’), definitely (‘the man entered’), rigidly (‘Tom’), deictically (‘this man,’ ‘he’), or personally (‘you,’ ‘I’). Beyond the noun phrase, we obtain forms of reference to *states*, then *events*, then propositions, then *facts*. It is a crucial claim of un-Cartesian linguistics that *all* of these forms of reference *co-vary* with specific grammatical configurations (Sheehan and Hinzen, 2011; Hinzen and Sheehan, 2013, Chap. 4; Martin and Hinzen, 2014); that no further forms of reference, in some non-linguistic world of thought, are known; and that in animal cognition (‘non-linguistic thought’), *none* of these forms of reference are seen (Hauser, 1996; Fitch, 2010). These findings together suggest that our language capacity *is* what avails us of these forms of reference; that when the limits of grammar are reached, these limits are the limits of thought as well; and that when grammar loses its grip on thought, thought must disintegrate^13^.

In organizing the forms of reference to the world, language negotiates both thought and reality while keeping them distinct. Every utterance we make involves a thought, which is internal, but it also has a ‘content’: it *ipso facto* says something *about the world*, which is *independent* of my thinking. In talking, we always both *maintain* and *cross* the divide between thought and the real. If this capacity is lost, which is descriptively the phenomenon we here seek to capture, the question *why* it is lost must lie in the question what it *is* to understand when a sentence such as ‘I am Jesus’ is true. We answer that it is not for it to *seem* to me that I am Jesus, or that I *want* to be Jesus, or that I *think* I am Jesus, or that it is *evident* for me that I am, or that *he* is Jesus. It is simply for me to be Jesus. But how do I know that these and only these are the right conditions for it to be true? From the *grammar* of the sentence, which is a copular clause that does not embed the content that I am Jesus under any such matrix verb as ‘seem,’ ‘want,’ or ‘think,’ and involves 1st Person self-reference. In this sense it is the grammar that tells me that I need to make a distinction between thought and reality, between what seems to be the case and what is. A loss of a grip on grammar in this way entails a loss of a sense of truth, predicting quasi-perceptual certainties to arise in language anomalously as well as ambivalence in Bleuler’s (1911) sense and stimulus distractibility: language *orders* our experience, and where this order fails, it makes sense that things will become salient that in grammatical cognition would simply be regarded as matters of ‘context’.

As noted, utterances not merely express thoughts but involve an implicit reference to our own 1st Person. All referential phrases within utterances require Person specifications that are fixed relatively to this 1st Person. Thus ‘he’ in ‘He sleeps’ encodes the 3rd Person Singular, in agreement with the inflection of the verb; its meaning is that the relation of the person referenced, who is the agent of the act of sleeping denoted, to the speech act participants, as and when the act takes place, is non-identity. Both the speech act and its 1st Person subject are thus implicitly referenced in Person distinctions^14^. And in thinking as such, insofar as it involves reference as well, the situation cannot be very different. As a result of the requirements of grammatical Person, all reference to the world in language takes place in a ‘triangular’ deictic space spanned by a threefold person distinction: *I*, the speaker and center of the deictic space, refer, in my speech act, to a person, non-identical to both me and *you*, my hearer, and to a fact about this person, which as such holds independently of us all, being a fact about the world (‘it’; see **Figure 1**).

Specification of Person is a ‘syntactic’ constraint on grammatical well formedness as well as propositionality in language: there is no truth in language without such deictic anchoring. Our proposal (**Figure 2**) is that all the positive symptoms above can be characterized as disturbances in this single grammatically determined deictic frame, which predicts symptoms depending on which of its corners is disproportionately affected. If it is not clear who speaks, speech perception can more easily become abnormal as in AVHs. Where the perceptual saliency of these deictically loosened thoughts is not high, the loosening itself is enough to lead to confusing experiences in which one doesn’t know whether the thoughts are mine, yours, or simply truths. Some may appear up for public viewing (*thought broadcast)*, or taken out of the speaker’s mind (*thought withdrawal*), who loses 1st Person control over them. In yet other cases, this same 1st Person finds itself thinking thoughts that are experienced as a 3rd Person’s (*thought insertion*). A ‘self-disturbance’ must be the result: for to understand the notion of self, we need all of the foregoing: concepts including a concept of thought, a capacity for reference, where acts of reference take place in a deictic frame marked by Person distinctions.

Phenomenological psychiatry set out to study a person’s subjective experience as a premise for a search for neurocognitive mechanisms giving rise to symptoms. Yet the distinctions that are lost in symptoms are *linguistic* ones. There is no more to the distinction between whether a given thought is my thought or yours or a third party’s than this very distinction between 1st, 2nd, and 3rd person. There is no more to the distinction between me being Jesus and me thinking that I am, than this very grammatical distinction between a clause that is embedded, functioning as a syntactic argument, and one that is not. I think about *the person* I am, my history, my body, my properties, or else just *me* – the thoughts are different, and distinction is the linguistic distinctions it is. ‘I’ cannot be equivalent to any of these other ways of referring to me, because ‘I’ encodes no description, and hence cannot be equivalent to one. If I start thinking of myself in a ‘third person kind of way’ (Sass, 2014), this is to *change* a mode in which I normally use linguistic distinctions to refer to myself. No known system other than language involves such distinctions, encoding propositionality, Tense, and Person in every utterance we make.

## The Language Profile of Schizophrenia Against our Predictions

We have now given a linguistic characterization of the positive symptoms, and we have a linguistic model that derives a propositional and referential capacity and new form of selfhood from language, suggesting that grammar (which critically includes Person) is the essential principle of organization of human-specific thought. A breakdown of language viewed this way *predicts* the loss of a propositional and referential capacity, and this loss is virtually *descriptively equivalent* to what we see in positive symptoms. There is no gap here between symptoms and the single essential explanatory variable invoked. Linguistic dimensions in these symptoms moreover do not involve elements peripheral to the organization of language or irrelevant to its role in the organization of thought, but concern its *primary function* as viewed in our linguistic framework, namely to convert conceptual knowledge (the mental lexicon) into *referential expressions* that have content on an occasion of use. Based on this, highly specific testable predictions arise, such as:

The more grammatically (and less lexically) mediated forms of reference become in language, the more reference will be impaired or anomalous. Splitting the forms of reference mentioned above into two halves, with generic and indefinite forms on one side, and definite, rigid, deictic, and personal ones on the other, the latter should be disproportionately affected. We do not know whether this is true, but note that:
(i) Delusional statements tend to be specific, involving the 1st person pronoun, proper names such as Jesus, Superman, or the Mafia; and by definition, they arise at the *sentential* level, where alone truth values are encoded.
(ii) Lexical knowledge as such (as measured in confrontational naming tasks), by contrast, should be least impaired of all, which coheres with evidence (McKenna and Oh, 2005). As noted, also neologisms and strange word uses arise within occurrences of them in grammatical frames, where they are referentially used.
(iii) Problems in encoding definite forms of reference are virtually a re-description of the symptom of ‘poverty of content,’ or ‘empty philosophizing’ used to characterize some forms of thought-disordered speech (Andreasen, 1979).
(iv) It is often noted that patients with schizophrenia ‘frequently fail to use pronominal reference correctly’ (Frith, 1992, p. 99; see also Rochester and Martin, 1979; Hoffman et al., 1985; McKenna and Oh, 2005, p. 112; Watson et al., 2012): pronouns are often used without their reference being clear to the listener, and they fail to track referents across discourse. Pronouns are the most grammaticalized form of reference that exist in language, and pronouns paradigmatically, and almost exclusively so in English, encode Person distinctions in grammar^15^. As Bleuler (1911, p. 118) reports, some patients speak of themselves only in 3rd Person, preferring to use their own name; one chronic patient only ever spoke in the 2nd.
(v) ‘Referential failures’ are highlighted in Ceccherini-Nelli and Crow (2003) as having distinctive diagnostic value. Vague references and missing information references are reported to be over-represented in schizophrenic patients, with referential disturbances transpiring as a trait-like features of the illness independent of symptom (or FTD) severity (Holzman et al., 1986; Docherty et al., 1988, 1996, 2003)^16^.

Frith (1992, p. 98), by contrast, refers to the ‘general consensus that only the highest levels of language processes are impaired in schizophrenia,’ by which it is intended that lexical, ‘syntactic,’ and phonological knowledge are relatively *unimpaired*, whereas processes of ‘discourse planning’ are affected, as words and sentences are combined with one another. However, there *is* no ‘discourse planning’ when there is no capacity to use language in the normal, i.e., the propositional way, with sentences configured that are true or false. A ‘pragmatic’ capacity to communicate in language and to maintain a discourse plan *presupposes* a grammatical ability to configure propositional information: by definition, pragmatics begins where grammar leaves off. Is this propositional capacity maintained in schizophrenia? Not in *any* of the positive symptoms, by definition of these symptoms; nor does the problem lie in communication, when the problem instead is that thought is heard, or the content of thought is the problem. If so, discourse is disordered because a propositional capacity is lost, not *vice versa*. A patient who answers ‘Yes, iron is heavy’ to ‘Are your thoughts wearing heavily on you?’ (McKenna and Oh, 2005, p. 104) fails at a discourse level. He hasn’t understood that ‘heavy’ was predicated (metaphorically) of thoughts – a sentence-level failure to integrate word meaning with the grammatical frames in which they occur.

‘Syntax’ cannot be unimpaired (Marini et al., 2008) in schizophrenia, if we view grammar as an inherent (rather than contingent) aspect of what sentences mean. In speech, grammatical complexity is never built without complexity in forms of reference and propositional meaning arising alongside. But these are impaired. Syntax moreover *is* empirically found to be impaired. Morice and Ingram (1982) achieved a differential diagnostic accuracy of 95% for schizophrenic vs. manic and controls based on a linguistic profile involving in particular a reduction in syntactic complexity, and syntactic and semantic errors, without distinguishing between FTD and other symptoms (see further Chaika and Alexander, 1986; Fraser et al., 1986; Hoffman and Sledge, 1988; King et al., 1990; Thomas et al., 1990; Oh et al., 2002; Walenski et al., 2009). Lower syntactic complexity includes lack of clausal embedding – which ipso facto entails that speech does not encode thinking about mental states or ToM: changes in grammatical complexity and patterns are not merely a ‘formal’ deficit, but have consequences for the meanings we grasp and convey. If propositionality, reference, and Person uniquely go with forms of grammatical organization, and to mediate them is language’s cognitive role, thought disorder can be investigated on a linguistic basis (Morice and McNicol, 1986, p. 248).

A number of studies found defects identified under the label ‘semantic,’ including semantic memory, i.e., abnormalities revolving around words, general knowledge, and concepts (Tamlyn et al., 1992; McKenna and Oh, 2005; Wang et al., 2011), which are language-related. Oh et al. (2002) found ‘expressive semantic’ anomalies to be characteristic of FTD independently of general intellectual impairment, yet crucially not in naming but only at a grammatical level of speech organization, where the referential function of language resides. The finding is consistent with what Kleist (1960) referred to as a ‘higher-level’ impairment, ‘responsible for word derivations, word constructions, the formation of sentences, and for the abstract meaning of speech conceptions – i.e., the thinking based on speech’: in other words, language impairment is at a level of language viewed as *integrated* with thought, or grammatical meaning as it is viewed in the un-Cartesian framework.

Just as ‘discourse coherence’ is a putatively non-linguistic variable that characterizes schizophrenic speech, the notion of ‘context processing’ has been prominently invoked (see McKenna and Oh, 2005, pp. 102–108). As with pragmatics, however, the notion of ‘context’ analytically presupposes that of ‘content.’ By defining propositional meaning, grammar on the present model delineates content, and co-defines context, while no other system is known to draw this distinction (Hinzen, 2015). If I hear the sentence ‘Mary smiles’ uttered, then it is a matter of context if she also wears red shoes, or the speaker has a hoarse voice. This distinction disappears (everything becomes context), if the sentence is not understood as a proposition that as such defines a notion of context as what is irrelevant to its content. A disturbance in a language-mediated propositional competence therefore predicts a disturbance in the understanding of context: while the patients generate speech syntactically, their ‘ability to organize verbal messages into meaningful grammatical units may be relatively fragile and subject to disruption’ (Hoffman et al., 1985, p. 199). Tone of voice or the sound form of a word then become significant and relevant to content, rather than being demoted or inhibited as part of the context, predicting distractibility and derailment. This is in line with results documenting difficulties of integrating word meaning with grammatical frames, as well as patients’ relative lack of sensitivity to grammatical constraints (Kuperberg et al., 1998, 2006). Where the boundary between content and context is shaken, speech will also fail to exhibit a literal-non-literal distinction, predicting the ‘concretism’ of schizophrenic speech.

Speech in FTD may *appear* to be grammatically normal and to be organized according to topics/subjects and comments/predicates. However, for there to be fluent speech at all, there cannot but be use of grammatical frames – the same ones that any patient with schizophrenia will have used for the first two decades of his life virtually incessantly. But when we investigate the linguistic profile of schizophrenia more closely, we see that what *appears* to be demarcated grammatically as the topic of conversation is not really *treated* as that: the patient derails, and topics can shift according to any association made. In a similar way, what *appears* to be a predicate applied to an argument yielding an apparent assertion with a truth value, e.g., the predicate ‘fell into the front doorway’ as applied to the subject ‘the pond,’ cannot really be that, for that a pond fell into a front doorway is nothing that anyone in his right mind could possibly state as being true. What appears to be a referential noun phrase such as ‘my spouse,’ as in ‘my spouse left,’ turns out not to be understood as such when we discover, a sentence later, that the patient refers to three spouses she has at the same time, depriving the phrase ‘my spouse’ in question of the required unique referent, and when we discover, again a sentence later, that the patient also thinks he has never been married, depriving the noun phrase of a referent altogether. Grammar is used, but it has lost its intrinsic meaning^17^.

## Language and the Cognitive Neuropsychology of Schizophrenia

By the 1980s, neuropsychological deficits in the schizophrenia population were well established, involving both general intellectual impairment (Liddle and Crow, 1984; Buhrich et al., 1988) and selective deficits, as in memory (especially verbal memory: McKenna et al., 1990; Saykin et al., 1991) and executive control, independently of general intellectual impairment (Laws, 1999; Barrera et al., 2004; Henry and Crawford, 2005). None of these cognitive functions, however, are in humans completely independent of language, which integrates them but also expands and transforms them, as compared with their forms in other species and hominins (Coolidge and Wynn, 2007). Nor is clear how neuropsychological deficits as seen in neurological patients should give rise to the positive symptoms, which as McKenna (2007) stresses are not properly said to be ‘deficits^18^.’ And while many correlations between neuropsychological test performance (especially executive dysfunction) and negative and disorganization symptoms exist (Liddle, 1987), this does not seem to be true for positive symptoms (Donohoe and Robertson, 2003; McKenna and Oh, 2005, Table 6.3; Dibben et al., 2008; Clark et al., 2010). In Dibben et al.’s (2008) meta-analysis, pooled correlations between executive impairment and negative/disorganization symptoms were small to moderate, while positive (‘reality distortion’) symptoms ‘close to zero.’ Berenbaum et al. (2008) found that AVHs correlate with abnormalities in episodic memory, which is consistent with our approach, while delusions were correlated with no neuropsychological dimension. The latter finding we also expect: delusions affect the content part (top vertex in **Figure 2**), not processing aspects (production/comprehension) to which standard neuropsychological tests may primarily be sensitive.

Frith (1992) broached new terrain by arguing that a number of positive symptoms could be explained by a specialized form of executive dysfunction implicated in the self-monitoring of willed actions, including acts of thinking. This theory held considerable promise in the domain of alien control symptoms and possibly also AVHs, but its support from imaging studies is less clear (McKenna, 2007, p. 254). In particular, in AVHs, contrary to what is predicted by the executive dysfunction theory, there is no de-activation of frontal areas (or other areas), but an increased activity in the temporal lobe neocortex, often bilaterally. By contrast, while listening to external speech, patients with AVHs show a decreased activation in the left superior temporal gyrus (including Wernicke’s area), which is fundamental for speech perception. This reversed pattern in comparison to controls suggests, as Plaze et al. (2006) put it, that ‘auditory hallucinations compete with normal speech for processing sites within the temporal cortex in schizophrenia.’ In line with this, hallucinators seem more prone to misattribute their own external recorded (and manipulated) speech than patients without AVHs and controls (Allen et al., 2007).

Generalized to include all ‘mentalizing’ (ToM), Frith’s (1992) theory appeared promising at a theoretical level with regards to persecutory and referential delusions as well, though it has less scope in the case of delusions of the ‘I am Jesus/Superman’ type. A recent review (Garety and Freeman, 2013), surveying 199 studies, concludes that with respect to delusions generally, ‘the ToM account has not stood up to subsequent testing.’ A further problem is whether ToM, like the psychological concept of ‘meta-representation,’ can be a promising candidate for a language-independent cognitive variable. As such Frith (1992) invoked it to explain disturbances in productive speech in FTD. The idea was that a defect in executive control would prevent a thought-disordered speaker to structure their discourse in accordance with a listener’s needs – a ‘pragmatic’ deficit. Hence there would be no language dysfunction *per se* in this syndrome, a view he saw supported by the putative lack of a comprehension deficit in language. However, a comprehension deficit has by now often been reported (Kuperberg et al., 1998, 2006; Condray et al., 2002; Tavano et al., 2008; Thoma et al., 2009). Nonetheless, ToM deficits in the wider schizophrenia phenotype, irrespective of symptoms, are well established by now (Frith and Corcoran, 1996; Bruene, 2005; Anselmetti et al., 2009; Bora and Pantelis, 2013). However, we know independently, from both normal and abnormally developing populations, that ToM is highly correlated with language in development (Astington and Jenkins, 1999; DeVilliers and Pyers, 2002; Hale and Tager-Flusberg, 2003; deVilliers, 2007; Newton and deVilliers, 2007; Paynter and Peterson, 2010). Whatever explanatory potential the ToM notion contains, therefore, a linguistic perspective may comprise it independently.

Hinzen and Sheehan (2013) argue that insofar as ToM denotes a conceptual rather than perceptual ability, language makes ToM redundant as a psychological construct, since it engenders our cognitive mind-reading ability (‘reading’ being indeed an appropriate metaphor). In particular, language competence entails the formation of what Frith (1992) termed ‘first-order representations,’ like ‘John is tall.’ The difference between this and a representation with ToM content, i.e., a ‘second-order’ or ‘meta’-representation, is simply the application of a grammatical operation: for the difference is not that between ‘John is tall’ and ‘John is sad,’ but like that between ‘John is tall’ and ‘Bill believes John is tall.’ The former distinction is a lexical one, the latter a grammatical one. If the latter was said to be as such a mental or non-linguistic one, it would still have to mirror the grammatical one exactly. The grammatical distinction is available to a mind the moment that argument-taking is an option – which is the moment that there is grammar. A grammar in which there are arguments can make clauses arguments, too, which then are embedded in other clauses. If it does, ToM content *ipso facto* arises, and no special capacity for representing mental state contents has to be postulated beyond grammar itself, which we need anyhow. A putative non-linguistic ToM module also does not as such entail any propositional capacity, and it fails to yield Person distinctions: ‘mentalizing’ is an unspecific term, and if it is propositional, this does not follow from anything in ToM. Crucially, second-order representations are propositions too: it can clearly be false that Bill believes that John is tall. Such representations are ipso facto part of language, which entails their propositionality for free.

In sum, invoking impairments in neurocognitive variables primarily known from neurological patients has met with considerable obstacles in schizophrenia. Correlations of variables with these symptoms have proven difficult; the mechanism of symptom formation is unclear; and core variables like ToM are not likely language-independent. This invites factoring language into the cognitive neuropsychiatry of schizophrenia and to develop more fine-grained clinical tests of grammar-based meaning distinctions to re-assess the connection between language and cognition.

## Language in the Schizophrenic Brain

Our account makes us expect the finding that neuroimaging the brain of persons with FTD in particular, have identified anomalies in classical language-related areas and circuitry (Weiss et al., 2005; DeLisi et al., 2006; Catani et al., 2011). Sans-Sansa et al. (2013) found an association of FTD with gray matter volume reductions in both Broca’s and superior temporal gyrus (including Wernicke’s area) along with ventromedial prefrontal cortex (vmPFC) and orbitofrontal cortices, the latter extended dorsally to the insula (see also Horn et al., 2010). The vmPFC is a classical ToM area (Saxe, 2006), and Ferstl et al. (2008) present results that ‘strongly suggest an overlap between the extended language network (ELN) and the regions implicated for ToM processes.’ Further findings indicate a convergence his suggests that relegating dimensions of language use to a ‘post-linguistic’ domain of ‘pragmatics’ may reflect classical conceptions of language driven by a separation of language from its use, which we here do not adopt. Hagoort and Levinson’s (2014) defense of the so-called ‘immediacy assumption’ may support this claim. between the ELN and the ‘language comprehension network’ of Turken and Dronkers (2011), which in turn strongly overlaps with the ‘(conceptual) semantic system’ of Binder et al. (2009), who sees the latter as ‘strikingly similar’ to the ‘default state’ of Binder et al. (1999) and Raichle et al. (2001), and further the ‘autobiographical memory retrieval system’ of Maguire (2001), Svoboda et al. (2006). And Pomarol-Clotet et al. (2010), finally, identify the mPFC ‘as a prominent site of abnormality in schizophrenia,’ connected to the default state through failures of deactivation, which the authors connect to conceptual over-activations mediating a sense of ‘self.’

Many studies have reported aberrant patterns in fronto-temporal networks across schizophrenia in response to a range of tasks with linguistic demands (Ngan et al., 2003; Kircher et al., 2005; Weinstein et al., 2006, 2007; Kuperberg et al., 2007, 2008; Dollfus et al., 2008; Borofsky et al., 2010). In addition, while normal adults exhibit left-lateralization of neural activity in fronto-temporal regions during language processing (Vigneau et al., 2006), individuals *across* the schizophrenia spectrum show more bilateral and right-lateralized activity during speech processing, verbal fluency, and lexical discrimination tasks (Weiss et al., 2005; Li et al., 2007; Diederen et al., 2010). Angrilli et al. (2009) found, for a sample of patients with positive and negative symptoms, and a high level of delusions – but scarce AVHs – difficulties concurrent with a ‘hemispherical indecision’ specific to phonological processing. This failure of left hemispheric dominance of phonology appears then to extend to schizophrenia in general, which suggests that *linguistic sound processing* is to some extent impaired throughout the disease.

## Conclusion

A linguistic model of positive symptoms may cast fresh light on their pathogenesis and underlying neuropsychology. ‘Thought’ is not a standard neurocognitive variable. This makes it hard to tackle delusional thought. If thought of the kind we see impaired in positive symptoms is language-mediated inherently, and a disintegration of basic functions of language in the configuration of reference is seen empirically in symptoms, then language could *be* a key neurocognitive variable to be targeted in understanding symptom formation, therapeutic intervention, and cognitive remediation.

## Conflict of Interest Statement

The authors declare that the research was conducted in the absence of any commercial or financial relationships that could be construed as a potential conflict of interest.

## Footnotes

1 Cf. Frith (1992, p. 97), who argues that there is a ‘fundamental difference between language and thought (…). Thinking is a private matter, whereas language is arguably the most important method we have for communicating with others.’

2 It builds on a long tradition in which language is regarded as primarily a tool for thought rather than communication (von Humboldt, 1836; Mueller, 1887; Chomsky, 2007).

3 Project ‘Language and Mental Health,’ Arts and Humanities Research Council grant AHL004070/1, 2014–2017.

4 Cf. Titone et al. (2007, p. 93): ‘the thinking anomalies associated with psychotic conditions are not, fundamentally, speech or language disorders (…). Rather, when language is used in an idiosyncratic way, it represents the outcome of a deviant thought process.’ This view has a long pedigree: ‘the causation of schizophrenic speech defect lies in an underlying thought-disorder rather than in a linguistic inaccessibility’ (Critchley, 1964, p. 359); also Maher (1972).

5 Consistent with this, Baethge et al.’s (2005) study of hallucinations among 4972 hospitalized persons confirms that the most frequent hallucinations were auditory, followed by somatic and visual hallucinations. Hallucinations in the schizophrenia group were comparatively the most severe and *least visual*. Subtypes of Auditory Verbal Hallucinations (AVHs) associated with the re-living of traumatic experiences in post-traumatic stress disorder, too, are said to be ‘usually visual, sometimes olfactory but rarely auditory verbal’ (McCarthy-Jones et al., 2014, p. S277).

6 All four *AVHs* subtypes identified in McCarthy-Jones et al. (2012) are identified by linguistic distinctions: in the first (commanding and commenting), the voice hearer is a 2nd- or 3rd-person addressee from the viewpoint of the voice who predominantly uses imperatives; the second (replays of memorized speech interactions) is no communicative experience as such; in the third, the voice speaks in the voice hearer’s own 1st person: in the fourth, there is a semantic deficit.

7 Bell (2013) suggests that AVHs are hallucinated acts of *communication*, reflecting a disturbance in ‘social cognition.’ A linguistic account of AVHs *entails* such a disturbance. The communication in question is crucially *linguistic*, and in normal conditions, perceiving speech *is* to perceive both acts of communication and agents behind them, while the reverse is not the case.

8 Critically, even an assertion like ‘I am sad’ is normally understood as telling us about something more than the mental state of the speaker, implicating a fact about the world – that the speaker is sad (lying is an exception and based on the fact here noted).

9 The first two have two 3rd Person referential expressions, while core delusions tend to involve the grammatical 1st Person. The third is generic (and 3rd personal). The fourth is finitely tensed. And as occurring embedded in a larger sentence like ‘I think I am Jesus,’ ‘I am Jesus’ expresses no delusion in the technical psychiatric sense.

10 Cf. Newen and Vogeley (2003): ‘In language, the correct assignment and involvement of 1PP [first person perspective] is reflected by the use of first-person pronouns.’ Clearly, forms of self-reference in the 1st Person could be constitutively involved in the formation of a ‘first person perspective.’ Its loss is reflected in pronominal shifts that have been widely attested in autism (Bartolucci and Albers, 1974; Lee et al., 1994; Hobson et al., 2010).

11 Its shortcomings have been exposed by one of the fathers of phenomenology himself, Merleau-Ponty (1964), who argued that language fundamentally *changes* our relations to the world and ourselves.

12 This linguistic derivation of self-disturbance accords with Mishara et al.’s (2014) conception of patients with ‘Ichstörung,’ who ‘(1) struggle to identify basic emotions in themselves, (2) grapple with distinguishing their own mental operations from others, (3) have difficulty viewing events from multiple perspectives, and (4) not make use of knowledge, in contrast to other groups, of their own thinking when facing challenges’ (see also Lysaker et al., 2010; Nelson et al., 2012). Patients facing such difficulties are *linguistic agents*, grappling with linguistic distinctions and seeking to determine what is real.

13 This predicts that insofar as core cognition is unimpaired in severe and global aphasia (Varley, 2014), the patients’ brain still somehow functions by the grammatical code that it has used every minute of six or so decades prior, and the difficulty lies in externalizing and perceiving this code in speech.

14 The speech act is referenced a second time in the grammatical encoding of *Tense*, which in this case encodes that the event denoted by the verb takes place as and when the speech act does, and is ongoing.

15 Pronouns are highly grammaticalized (Martin and Hinzen, 2014) and in this sense not a matter of ‘discourse-cohesion’ in some non-linguistic sense.

16 Symptoms like peculiar use of language, disorganized and disconnected speech, and verbal underproductivity, which are more state-dependent in mania and frequently involve a return to normal levels during clinical remission (Andreasen and Grove, 1986; Harrow and Marengo, 1986; Spohn et al., 1986), are found to be present in schizophrenia in residual forms during both acute episodes and remission (see also Harvey et al., 1984, 1990; Marengo and Harrow, 1997).

17 This point arguably extends to the simplest forms of what is *described* in the literature through the psychological term ‘conceptual combination.’ Titone et al. (2007) show that patients with schizophrenia ‘combine concepts’ differently. Hinzen and Sheehan (2013, Chap. 8) argue that the best predictor for their performance is in fact a grammatical deficit. Moreover, ‘concepts’ of a non- or pre-linguistic kind (e.g., Carey, 2009) are not known to productively combine in the way of words.

18 The explanatory problem is analogous to the one arising from positing deficits in perception: how would these give rise to delusions, say, when misperceiving typically does not have this effect?
